# Relationship between Interdialytic Weight Gain and Blood Pressure in Pediatric Patients on Chronic Hemodialysis

**DOI:** 10.1155/2016/5972930

**Published:** 2016-10-30

**Authors:** Olivera Marsenic, Michael Anderson, Kevin G. Couloures

**Affiliations:** ^1^Pediatric Nephrology, Yale University, New Haven, CT, USA; ^2^Biostatistics and Epidemiology, University of Oklahoma Health Sciences Center, Oklahoma City, OK, USA; ^3^Pediatric Critical Care, Yale University, New Haven, CT, USA

## Abstract

Overhydration is reported to be the main cause of hypertension (HTN) as well as to have no association with HTN in hemodialysis (HD) population. This is the first report of the relationship between interdialytic weight gain (IDWG) and pre-HD blood pressure (BP) in pediatric patients in relation to residual urine output (RUO). We studied 170 HD sessions and interdialytic periods performed during a 12-week period in 5 patients [age 4–17 years, weight 20.8–66 kg, 3 anuric (102 HD sessions), and 2 nonanuric (68 HD sessions)]. BP is presented as systolic BP index (SBPI) and diastolic BP index (DBPI), calculated as systolic or diastolic BP/95th percentile for age, height, and gender. IDWG did not differ (*P* > 0.05) between anuric and nonanuric pts. There was a positive but not significant correlation between IDWG and both pre-HD SBPI (*r* = 0.833, *P* = 0.080) and pre-HD DBPI (*r* = 0.841, *P* = 0.074). Pre-HD SBPI (1.01 ± 0.12 versus 1.13 ± 0.18) and DBPI (0.92 ± 0.16 versus 1.01 ± 0.24) were higher in nonanuric patents (*P* < 0.001 and *P* < 0.01, resp.). Pre-HD HTN may not be solely related to IDWG and therapies beyond fluid removal may be needed. Individualized approach to HTN management is necessary in pediatric dialysis population.

## 1. Introduction

Role of interdialytic weight gain (IDWG) in hypertension (HTN) is poorly defined. While some report overhydration as the main cause of HTN, others find that it is frequently not associated with HTN, in both adults and children [1–4]. Fluid removal by ultrafiltration (UF) during hemodialysis (HD) is commonly the first-line therapy used to manage HTN in dialysis patients. However, aggressive fluid removal may lead to intradialytic hypotension, with reduction of blood flow to vital organs [5, 6], and should be avoided if HTN is not caused solely by fluid retention. Relationship between IDWG and blood pressure (BP) may vary between children of different age and size and may be affected by growth and associated variations in total body water, dry weight (DW), and necessary fluid intake to meet nutritional needs, as well as nonadherence to medications and fluid restriction. Therefore an individualized approach to management of fluid retention and HTN in pediatric patients is necessary. Although several studies investigated how IDWG relates to BP in dialyzed children, the effect of residual urine output (RUO) on IDWG and pre-HD BP in this population has not been studied [1–3, 6]. This is the first report of different relationships between IDWG and pre-HD BP in relation to RUO in pediatric patients (pts) on chronic HD.

## 2. Patients and Methods

The study was approved by Yale University Human Investigation Committee and DaVita Clinical Research® (Minneapolis, MN, USA). Hemodialysis was performed at the outpatient DaVita Dialysis Unit (DaVita HealthCare Partners Inc., Denver, CO, USA), affiliated with Yale New Haven Children's Hospital. This is a retrospective, longitudinal study, with repeated observations obtained from the same pt. Informed consent was not required. To be included in the study, patients needed to be stable (without hospitalization, changes in dialysis prescription, or addition of new medications) and treated with the same and constant dialysate sodium (dNa) concentration (138 mEq/L) during a standardized period of 12 weeks, allowing steady state in BP and fluid balance. Patients received three-times-weekly standard HD using Fresenius 2008K machines (Fresenius Medical Care, Waltham, MA).

We analyzed data collected as part of routine clinical care. Data collected and/or calculated included the following: HD characteristics, BP before and after HD, weight (Wt) before and after HD, estimated dry weight (EDW), ultrafiltration, IDWG [3], and monthly spKt/V [9].

BP was measured by pediatric dialysis nurse using the measuring device that is part of the dialysis machine with a size-appropriate cuff in sitting position [10] and was verified with manual BP monitor as appropriate. HTN was defined as pre-HD systolic BP (SBP) ≥ 95th percentile for age, height, and gender [10]. Pts were instructed not to take antihypertensive dose scheduled prior to HD.

EDW was continuously assessed and adjusted at least monthly, with routine use of intradialytic blood volume monitoring in correlation with clinical outcomes (intra- or interdialytic symptoms, intradialytic hypotension, and the lowest tolerated post-HD Wt) [3, 6]. Echocardiogram (ECHO) was routinely done prior to start of maintenance HD and at least annually on a nondialysis day. A single most recent ECHO performed prior to the 12-week study period was used for left ventricular mass assessment in each patient [7, 8]. A renal nutritionist provided education on sodium avoidance to all pts, without specifying the limit for sodium intake. Dietary recommendations did not change during the study period. Fluid restriction is routinely recommended for each patient based on estimated insensible fluid losses, RUO, and allowed IDWG of <5% of EDW. RUO was assessed every 6 months with a 24 hr urine collection.

Data are presented using descriptive statistics and box plots. Correlation analysis was performed using Pearson correlation coefficients and corresponding *P* values. Comparisons were made using Student's *t*-test.

## 3. Results

Total of 170 HD sessions with corresponding interdialytic periods were studied in relation to RUO (*n* = 102 HD sessions in anuric group; *n* = 68 HD sessions in nonanuric group) in five pts that fulfilled inclusion criteria. Equal number of HD sessions per patient (34/pt) were studied. Three pts were anuric (pts 1, 2, and 3) and 2 were nonanuric (pts 4 and 5). All subjects had normal cardiac function with left ventricular mass (LVM) index (gr/m^2.7^) percentiles 50 to 99 [7] and LVM relative to lean body mass percentiles 50 to >97 [8]. Mean time from LVM assessment to start of study was 5.6 months. During the 12-week study period, pt 5 required increase in antihypertensives, while antihypertensives were not changed in the remaining 2/3 pts receiving them (Table 1).

**Figure 1:**
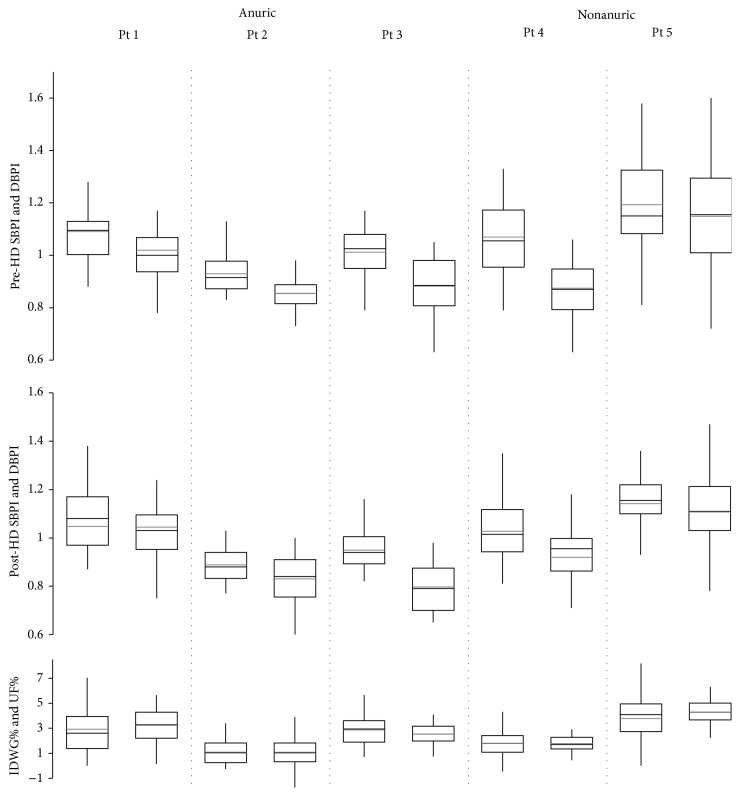
Interdialytic weight gain (IDWG) and ultrafiltration (UF) and pre-HD and post-HD systolic blood pressure index (SBPI) and diastolic blood pressure index (DBPI) presented per patient. SBPI and DBPI are presented as left and right boxplot, respectively. IDWG and UF are presented as left and right boxplot, respectively. Boxplot: the box represents the interquartile range (Q1–Q3) with gray line representing the mean and black line representing the median; whiskers extend to 1.5 of the interquartile range.

Results are presented in relation to RUO in Tables 1 and 2 and Figure 1. We found significantly higher pre-HD SBPI and DBPI in nonanuric pts in comparison to anuric pts (*P* < 0.001 and *P* < 0.01, resp.). IDWG was higher in nonanuric pts (2.78 ± 2.38%) in comparison to anuric pts (2.30 ± 1.79%), but this difference was not statistically significant (*P* = 0.06) (Table 2). Using mean values for each pt, we found positive but not statistically significant correlations between IDWG and SBPI (*r* = 0.833, *P* = 0.080) and IDWG and DBPI (*r* = 0.841, *P* = 0.074). Individual data analysis showed different relationships between IDWG and pre-HD BP (Figure 1); 3/5 pts (pts 1, 3, and 5) had relatively higher pre-HD BP with large IDWG, 1/5 pts (pt 2) had relatively lower pre-HD BP with low IDWG, and 1/5 pts (pt 4) had relatively higher pre-HD BP with low IDWG. Post-HD SBPI decreased with fluid removal in all patients, with a better response to UF in anuric (*P* < 0.01) than in nonanuric (*P* < 0.05) pts. Both SBPI and DBPI remained higher (*P* < 0.001) after HD and UF in nonanuric pts in comparison to anuric pts.

## 4. Discussion

We found a positive association between IDWG and pre-HD BP. This is in agreement with prior studies where fluid retention was reported to be the main determinant of HTN but contrary to those that found it was not related to HTN [1, 4]. Paglialonga et al. retrospectively analyzed 16 oligoanuric children and found significant correlation between IDWG and left ventricular mass index (LVMI) with significant correlation between IDWG and DBP [3]. Haskin et al. performed 44 hr ambulatory blood pressure monitoring (ABPM) on 13 pediatric HD patients and found that with longer monitoring (1 day versus 2 days) more pts were found to be hypertensive, with moderate positive correlation between BP and IDWG, concluding that as fluid accumulates during the interdialytic period it contributes to increased BP load [2].

Efforts to decrease IDWG include decreasing dNa and dietary salt intake. Patients studied here were dialyzed using dNa of 138 mEq/L shown to result in lower IDWG than dNa 140 mEq/L [11]. To potentially further decrease IDWG by decreasing Na load and thirst, even lower dNa may be needed.

Relationship between RUO, IDWG, and pre-HD BP has not been studied in pediatric HD. Since fluid retention is thought to be the main cause of HTN in HD pts, RUO is assumed to be protective from high IDWG and pre-HD HTN. However, results from our nonanuric pts suggest the opposite. In comparison to anuric pts, our pts with RUO had significantly higher pre-HD BP without significant difference in IDWG. In addition, although post-HD SBP decreased with fluid removal in both anuric and nonanuric pts, the response to UF was better in anuric pts. Post-HD BP remained higher in nonanuric pts. Paglialonga et al. reported that, in their study of 16 oligoanuric children on HD, the 9 that had RUO showed no difference in IDWG in comparison to 7 anuric subjects. The authors did not study the effect of RUO on BP [3]. The relationship between IDWG and pre-HD HTN was different in the 2 pts with RUO in our study. Pt 5 had consistently very large IDWGs and severe pre-HD HTN, suggesting that pts with RUO are at the same risk of large IDWG as pts without RUO. Pt 4 had significant pre-HD HTN with relatively mild IDWG, suggesting that mechanisms in addition to fluid retention are responsible for pre-HD HTN. This is in agreement with findings of Zaloszyc et al. who retrospectively analyzed hydration status of 23 pediatric HD pts using bioimpedance spectroscopy and found that overhydration was present in only 32.3%, with majority of those that were overhydrated having normal BP. The authors reported that 24% of underhydrated and 45.3% of normohydrated pts had HTN and concluded that HTN is not always related to overhydration; 5/23 pts in this study had RUO, but its effect on BP was not analyzed [1].

Mechanisms responsible for HTN in dialysis pts include sodium retention with increase in extracellular volume, sympathetic activation, renin-angiotensin-aldosterone system activity, and abnormalities in properties of the vasculature [4, 12]. In addition, Na can worsen HTN by its effect on endothelial cell function and vasoregulators and by increasing peripheral vascular resistance due to sodium-triggered release of systemic and local vasoconstrictors [4, 11]. Risk factors for HTN in pediatric HD include black race, young age, acquired cause of renal failure, HTN prior to start of HD, and short time on HD with lack of adjustment to fluid and salt restriction [4, 12]. Our findings highlight the importance of salt and fluid restriction even in patients with RUO, as well as the need for therapies beyond fluid removal as indicated.

Similarly to other studies in pediatric hemodialysis, our study has disadvantages that include small number of subjects, retrospective design, and single center experience [2, 3, 11]. As only retrospective data that were part of routine clinical care were collected, our study lacks dietary salt intake records, ambulatory blood pressure monitor studies, 3 or more BP measurements before HD, daily urine output, daily weight, and ambulatory BP measurements. However, our study and its design have several advantages: this is the first study to assess the relationship between IDWG and pre-HD BP in relation to RUO, we analyzed data from a relatively large number of events (170 HD sessions), study period was standardized to 12 weeks for each subject with repeated observations from the same patient over time, and dNa was the same and constant for all subjects.

The patients presented here exemplify the different relationships between IDWG and pre-HD HTN that can occur in children and adolescents on HD. Pre-HD HTN may not be solely related to IDWG and therapies beyond fluid removal may be needed. Further studies are needed to investigate the relationship between IDWG and HTN, as well as etiology of HTN in children on maintenance HD with and without RUO. Development of individualized strategies for HTN management is necessary in pediatric HD population.

## Figures and Tables

**Table 1:** Patient (pt) and hemodialysis (HD) characteristics.

Characteristics	Pt 1	Pt 2	Pt 3	Pt 4	Pt 5
Age (years)	17	15	14	17	4
Primary diagnosis	FSGS	Kawasaki disease, MAS	FSGS, failed transplant	Renal dysplasia, failed transplant	FSGS
Race/gender	Black/F	Hispanic/M	Black/F	Black/F	Black/M
HD vintage (months)	39	11	138	7	9
Scheduled antihypertensives	Clonidine Nifedipine	0	0	Labetalol Amlodipine	Lisinopril Amlodipine
Other medications that may contribute to HTN	Norethidrone	Hydrocortisone	Norethidrone	Tacrolimus	—
LVMI percentile (gr/m^2.7^) [7]	90	50	90	95–99	95–99
LVM percentile relative to lean body mass [8]	90	50–75	90	75	>97
Urine output (mL/day/1.73 m^2^)^$^	Anephric	Anuric	Anephric	420	800
Mean *Kt*/*V* ^#^ [9]	1.98	2.05	1.80	1.47	1.84
Mean HD duration (min)	233	197	181	185	222
Mean IDWG%	2.93	1.09	2.87	1.79	3.78
Mean EDW (kg)	60.5	38.5	41.2	67.1	21.8

HTN: hypertension; FSGS: focal segmental glomerulosclerosis; MAS: macrophage activation syndrome; HD: hemodialysis; LVMI: left ventricular mass (LVM) index; M: male; F: female; EDW: estimated dry weight (wt); IDWG: interdialytic wt gain (Wt before − Wt after preceding HD); and IDWG%: IDWG expressed as % of EDW [3].

^$^Assessed routinely every 6 months with 24 hr urine collection.

^#^Blood flow rate 170–400 mL/min, dialysate flow rate 600 mL/min, and dialysate Na 138 mEq/L.

**Table 2:** Comparisons in relation to residual urine output, *n* = 170 hemodialysis (HD) sessions, and interdialytic periods; mean ± SD.

	Anuric (*n* = 102)	Nonanuric (*n* = 68)	*P*
Pre-HD SBP	126 ± 15	133 ± 19	*P* < 0.05
Pre-HD SBP index^*∗*^	1.01 ± 0.12	1.13 ± 0.18	*P* < 0.001
% of PreHD SBP > 95th percentile^*∗*^	52%	78%	—
Pre-HD DBP	75 ± 14	78 ± 16	n.s.
Pre-HD DBP index^*∗*^	0.92 ± 0.16	1.01 ± 0.24	*P* < 0.01
Post-HD SBP	120 ± 21	128 ± 15	*P* < 0.05
Post-HD SBP index	0.96 ± 0.16 (*P* < 0.01^#^)	1.08 ± 0.14 (*P* < 0.05^#^)	*P* < 0.001
Post-HD DBP	73 ± 15	78 ± 12	*P* < 0.05
Post-HD DBP index	0.89 ± 0.17 (n.s.^#^)	1.01 ± 0.19 (n.s.^#^)	*P* < 0.001
UF, %	2.30 ± 1.51	3.07 ± 1.69	*P* < 0.01
UF, kg	1.14 ± 0.86	1.07 ± 0.41	n.s.
IDWG, %	2.30 ± 1.79	2.78 ± 2.38	n.s. (*P* = 0.06)
IDWG, kg	1.15 ± 0.99	1.09 ± 0.66	n.s.

n.s.: not significant (*P* > 0.05). ^#^Comparison with pre-HD result in the same group.

HD: hemodialysis; SBP: systolic blood pressure (mmHg); DBP: diastolic blood pressure (mmHg); UF: ultrafiltration (Wt before − Wt after), UF%: UF expressed as % of estimated dry wt (EDW); IDWG: interdialytic weight (wt) gain (Wt before − Wt after preceding HD); and IDWG%: IDWG expressed as % of EDW [3]. ^*∗*^SBP or DBP/95th percentile for age, height, and gender [10].
